# The Historical Influence of Psychoanalytic Concepts in the Understanding of Brain Injury Survivors as Psychological Patients

**DOI:** 10.3389/fpsyg.2021.703477

**Published:** 2021-08-20

**Authors:** Christian Salas

**Affiliations:** ^1^Center for Human Neuroscience and Neuropsychology, Faculty of Psychology, Diego Portales University, Santiago, Chile; ^2^Clinical Neuropsychology Unit, Faculty of Psychology, Diego Portales University, Santiago, Chile; ^3^Psychodynamic Psychotherapy Unit, J.H. Barak Psychiatric Institute, Santiago, Chile

**Keywords:** brain injury, neuropsychological rehabilitation, psychoanalysis, psychotherapy, history

## Introduction

Since the early work of Goldstein (1954, 1995), psychoanalysis has been an influential theoretical and clinical perspective in comprehending our patients' emotional adjustment after brain injury. This, even though psychoanalytic mainstream has considered for decades “organic” patients as the paradigmatic example of contraindication (Cooper and Alfillé, 2011). The relationship between psychoanalysis and neuropsychological rehabilitation, the discipline specialized in helping brain injured survivors adjusting to physical, cognitive and behavioral problems, has been equally complex (Salas, 2014). Leading authors of the field have questioned whether psychoanalytic psychotherapy is suitable for this population and whether unconscious processes and early relationships have any relevance in rehabilitation (Wilson, 2014).

In this opinion article I will argue that psychoanalysis has strongly influenced the way in which psychologists, clinical neuropsychologists and rehabilitation professionals understand brain injured survivors as patients with important psychological rehabilitation needs. I will briefly summarize four key psychoanalytic ideas that have contributed to the development of neuropsychological rehabilitation.

## Four Key Psychoanalytic Ideas In Neuropsychological Rehabilitation

### Brain Injury as a Loss of Meaning in Life

This may seem an obvious idea today in neuropsychological rehabilitation, but this was not the case prior to the 90s, when rehabilitation was primarily focused on cognitive remediation and work productivity. At that time, George Prigatano, a clinical neuropsychologist influenced by Kurt Goldstein, Yehuda Ben-Yishay and Carl Jung, observed that brain injured survivors commonly reported existential problems related to the “loss of normality” (Prigatano, 1999). Interestingly, and using psychoanalytic insights, Prigatano argued that such losses should be understood from two perspectives. As a loss of the possibility to fit into societal standards, cultural notions, or *archetypes*—in the Jungian sense—regarding what is considered desirable and valuable in human beings (beauty, intelligence, success). As a consequence, Prigatano posited that psychological interventions for brain injured survivors should help them explore new cultural symbols in order to find a place in the world and rebuild meaning in life (work, love and play).

The loss of normality implied a loss of biographical normality as well, often portrayed by survivors as the longing “to be the one I was before.” Prigatano argued that we could not understand such biographical loss without considering psychodynamic factors such as defenses and early relationships with attachment figures (Prigatano, 2008; Salas and Prigatano, 2018). Accordingly, Prigatano proposed that “the one I was before,” and “the one I am now” were heavily defined by people's psychohistory. Our self-concept, what we consider as valuable and desirable in us, or what we dislike about us and hide from others, is the crystallization of many explicit and implicit interactions with those that cared for us. Thus, we should include psychodynamic factors in case formulations, and consider them when designing rehabilitation interventions. Otherwise, we will not understand situations where patients don't collaborate or don't adhere to interventions that are, from a clinician's perspective, obviously beneficial for them. Prigatano also observed that, due to the many cognitive impairments (loss of abstraction, cognitive inflexibility, language impairments, etc.) survivors often struggle understanding what they have lost, thus compromising the process of finding new existential meaning. He argued that rehabilitation should adapt psychological tools to bypass these impairments and facilitate meaning reconstruction after the injury. Furthermore, he extensively explored the use of symbols and metaphors to help survivors expressing and sharing their subjective experience (Prigatano, 2012). According to Prigatano, the “Journey of the Hero,” a classic Jungian archetype, proved to be especially useful tool to explore and rebuild meaning.

### Brain Injury as a Narcissistic Injury

It is interesting to note that Heinz Kohut used the cognitive consequences of brain damage to develop his conceptualization of narcissism. In *Thoughts on Narcissism and Narcissistic Rage* (Kohut, 1972) he described that the inability to find words, or the loss of control over our thinking processes after brain damage, can be experienced by survivors as the loss of a part of the self, triggering intense feelings of anger. To Kohut humans have a healthy, omnipotent relationship with their minds. We control our minds. We think and words emerge. We want something and our limbs reach for it. This healthy omnipotence gives us a sense of coherence between desires, thoughts and actions. According to Kohut, such omnipotence is at the base of a healthy self-esteem. After brain injury, cognitive and physical impairments often fracture the omnipotent relationship with the mind/body. In fact, a mind-body disconnection has been described as one of the most important changes faced by survivors after the injury (Levack et al., 2010).

The use of Kohutian ideas came into mainstream neuropsychological rehabilitation thanks to the work of Pamela Klonoff and colleagues. Klonoff, a clinical neuropsychologist and close colleague of Prigatano, proposed that brain damage could be experienced by some survivors as a narcissistic injury (Klonoff and Lage, 1991; Klonoff et al., 1993). This experience was thought to be particularly problematic in survivors with early relational traumas, who had not adequately developed a healthy narcissism due to environmental failures, where carers have not mirrored or re-affirmed their sense of self-worth. In these cases, a constant effort to appear perfect to oneself and others may take place—as a grandiose Self—in order to regulate a profound sense of shame that is attached to the belief that deep down there is something defective about the self. In these cases, the injury can fracture this defensive grandiose image, and primitive feelings of emptiness, worthlessness may re-emerge. Klonoff has described in detail how feelings of shame can be externalized as rage toward relatives or rehabilitation professionals and/or the self. These feelings may be so unbearable for the survivor that eradicating the self completely can work as a way of wiping out the offending, disappointing reality of feeling damaged. Kohut and Klonoff's ideas are of particular relevance in the psychological care of patients with past early traumatic histories. However, the loss of coherence between desires, thoughts, and actions, is a common experience amongst survivors, which needs acknowledgment and elaboration.

### Brain Injury Can Change the Dynamics of Emotion and Personality

Following the observation of long-term psychoanalytic treatments of brain injured survivors, Kaplan-Solms and Solms (2002) reported in their seminal book Clinical Studies in Neuropsychoanalysis that brain damage did not only alter the cognitive architecture of the mind (e.g., perception, language, memory, thinking), but more importantly, could change emotion, personality and motivation. This may seem an obvious fact today, but again, it was not at the time, now 20 years ago. The work of these authors has motivated a new generation of clinicians to use a similar approach to explore emotion, personality and motivation changes in a wide range of complex neuropsychological syndromes, previously often left out of psychological care, such as profound amnesia, confabulation and emotion dysregulation (for a review see Salas et al., 2021). Clinical Studies in Neuropsychoanalysis also refuted several taboos, with perhaps the most important one, that people with brain damage could not benefit from psychodynamic psychotherapy. Due to the influence of this book the field of neuropsychoanalysis emerged and clinical neuropsychologists began to include psychodynamic ideas in case formulations, as well as more routinely use psychodynamic tools in psychological treatments.

Clinical Studies in Neuropsychoanalysis did not only help neuropsychological rehabilitation to realize that there were dynamic changes in emotion and personality after brain damage, but also influenced psychoanalysis itself, by reviving Freud's *Project for a Scientific Psychology*. Such revival was welcomed by some, but simultaneously resisted by those who argued that psychoanalytic practice does not need a material theory of the mind/brain (see the “Case against neuropsychoanalysis” debate Blass and Carmeli, 2007). However, beyond the debate about the clinical suitability and usefulness of neuroscientific insights in clinical practice, Clinical Studies put on the table a revolutionary idea: meta-psychological concepts related to the dynamics of affect and personality had a neuroanatomical correlate. For example, lesions to specific cortical areas could modify the interactions between id, ego and super-ego. Such findings set the scene to new research programs exploring the affective nature of consciousness, the neural basis of drives and the interaction between affect and cognition (Fotopoulou et al., 2012).

### Brain Injury as a Loss of the Meeting of the Minds

In the last decade there has been an increased interest in the use of relational ideas to comprehend psychological and interpersonal changes after brain injury, as well as to develop psychological interventions that could potentially address these. Psychoanalysis has contributed to this development in many ways. Initially, and thanks to the work of Pepping (1993) and Lewis (1999), concepts like transference and countertransference were proposed as relevant to understand the development of a therapeutical, or adversarial, alliance between patients, clinicians, and the rehabilitation team. It was described, for example, that different types of countertransference could be experienced by professionals, thus expanding classic theorization on countertransference: to patients' experience, to their deficits or even to their attitudes toward their deficits. The relevance of counter transferential feelings has been highlighted by researchers reporting that negative emotions (frustration, fear, anger) are common amongst rehabilitation professionals (Judd and Wilson, 2005). Transference has also been referred to as a useful concept. In 1954 Kurt Goldstein proposed the active use, and promotion, of positive transferential feelings (trust) to engage survivors in a treatment they do not always understand completely. More recently, it has been reported that feelings toward parental figures can be transferred to rehabilitation professionals (Yeates and Salas, 2020) and that specific cognitive impairments—confabulation, amnesia—can shape and influence the transferential process (Tiberg, 2014; Moore et al., 2017).

Relational ideas appeared in neuropsychological rehabilitation around the 90s and 00s with two key psychoanalytic papers that emphasized brain injury as a *relational* loss. Feigelson (1993), portrayed in a deeply moving personal article the impact that brain injury had on the survivor-beholder (a partner, a sibling), who experienced the changes asociated to TBI as a personality death: the emergence of someone that looks like the person, but feels like a stranger. Clearly here the emphasis is not in the intrapsychic changes generated by the injury, but its *impact* in the relational space that defines, and emotionally coordinates, members of a dyad. Later, Freed (2002) developed Feigelson's ideas further by stressing how brain injury, and the anxieties generated by personality change in the partner, disrupted the experience of connection and attunement: the meeting of the minds. This generated a complex problem. After the injury, the survivor needs the mind of the other as a source of external cognitive and emotional regulation (auxiliary ego function). However, due to the anxieties generated by the experience of relating to someone who felt like a stranger, the other struggles in providing cognitive, practical and emotional support. These ideas were later influential to Yeates and colleagues, who proposed a relational (epistemological) turn in neuropsychological rehabilitation. In their book *A Relational Approach to Rehabilitation* (Bowen et al., 2010) they argue that brain damage does not occur inside people's skulls, but in the space between people, often infiltrating and amplifying personal distance and disconnection. Thus, brain injury and its socio-emotional consequences can be understood as socially constructed and modulated by social context. These ideas have inspired clinicians to develop psychological interventions using a relational (attachment-based) framework (Yeates and Salas, 2020).

## Discussion

The main goal of this article was to review key psychoanalytic ideas that have influenced the understanding of brain injury as a psychological problem, and of brain injury survivors as patients with unique psychological rehabilitation needs. Brain injury represents to many survivors a loss of normality, demanding the reconstruction of meaning in life. Subjectively, brain injury can be experienced as a loss of coherence, compromising the sense of control over our own minds and bodies. Brain damage can alter the dynamics of emotion and personality, as well as the relational space where minds encounter each other. Despite the historical and clinical relevance of these ideas, psychoanalytic concepts and techniques disappointingly are rarely included in training programs for clinical neuropsychologists. At present, this situation appears to be related to the unfamiliarity of many professionals with the evolution of psychoanalytic psychotherapies and its actual contribution to the treatment of mental health problems. Another relevant factor relates to changes across the globe in health care systems, which have demanded briefer and less expensive forms of psychological care. This shift has influenced the type of support provided by clinicians, with a predominant emphasis on symptom remediation (Salas and Prigatano, 2018). The intention of this article has been to sketch a brief genealogy of psychoanalytic ideas in neuropsychological rehabilitation, a genealogy to which younger generations can identify and contribute with their work.

## Author Contributions

The author confirms being the sole contributor of this work and has approved it for publication.

## Conflict of Interest

The author declares that the research was conducted in the absence of any commercial or financial relationships that could be construed as a potential conflict of interest.

## Publisher's Note

All claims expressed in this article are solely those of the authors and do not necessarily represent those of their affiliated organizations, or those of the publisher, the editors and the reviewers. Any product that may be evaluated in this article, or claim that may be made by its manufacturer, is not guaranteed or endorsed by the publisher.
